# Conserved repertoire of orthologous vomeronasal type 1 receptor genes in ruminant species

**DOI:** 10.1186/1471-2148-9-233

**Published:** 2009-09-15

**Authors:** Hiromi Ohara, Masato Nikaido, Atsuko Date-Ito, Kazutaka Mogi, Hiroaki Okamura, Norihiro Okada, Yukari Takeuchi, Yuji Mori, Kimiko Hagino-Yamagishi

**Affiliations:** 1 Laboratory of Frontier Science, Tokyo Metropolitan Institute of Medical Science, Tokyo, Japan; 2 Graduate School of Bioscience and Biotechnology, Tokyo Institute of Technology, Yokohama, Japan; 3 Laboratory of Veterinary Ethology, The University of Tokyo, Tokyo, Japan; 4 Laboratory of Neurobiology, National Institute of Agrobiological Sciences, Tsukuba, Ibaraki, Japan

## Abstract

**Background:**

In mammals, pheromones play an important role in social and innate reproductive behavior within species. In rodents, vomeronasal receptor type 1 (V1R), which is specifically expressed in the vomeronasal organ, is thought to detect pheromones. The V1R gene repertoire differs dramatically between mammalian species, and the presence of species-specific V1R subfamilies in mouse and rat suggests that V1R plays a profound role in species-specific recognition of pheromones. In ruminants, however, the molecular mechanism(s) for pheromone perception is not well understood. Interestingly, goat male pheromone, which can induce out-of-season ovulation in anestrous females, causes the same pheromone response in sheep, and vice versa, suggesting that there may be mechanisms for detecting "inter-species" pheromones among ruminant species.

**Results:**

We isolated 23 goat and 21 sheep intact V1R genes based on sequence similarity with 32 cow V1R genes in the cow genome database. We found that all of the goat and sheep V1R genes have orthologs in their cross-species counterparts among these three ruminant species and that the sequence identity of V1R orthologous pairs among these ruminants is much higher than that of mouse-rat V1R orthologous pairs. Furthermore, all goat V1Rs examined thus far are expressed not only in the vomeronasal organ but also in the main olfactory epithelium.

**Conclusion:**

Our results suggest that, compared with rodents, the repertoire of orthologous V1R genes is remarkably conserved among the ruminants cow, sheep and goat. We predict that these orthologous V1Rs can detect the same or closely related chemical compound(s) within each orthologous set/pair. Furthermore, all identified goat V1Rs are expressed in the vomeronasal organ and the main olfactory epithelium, suggesting that V1R-mediated ligand information can be detected and processed by both the main and accessory olfactory systems. The fact that ruminant and rodent V1Rs have distinct features suggests that ruminant and rodent V1Rs have evolved distinct functions.

## Background

Pheromones are chemical substances that are secreted externally by an individual and received by other individuals of the same species, in which they induce a specific behavior and/or neuroendocrine responses in receiving individuals [1-4]. In mammals, pheromones are mainly detected by the vomeronasal organ (VNO) [5-8]. The vomeronasal receptor type 1 (V1R) [9,10] and V2R [11-13] families are specifically expressed in sensory neurons of the rodent VNO. Both receptor types belong to the seven-transmembrane domain G protein-coupled receptor family, but they share no sequence similarity. Thus, these receptor types are generally considered to have evolved independently. The deletion of 16 mouse V1R genes results in altered social behavior and loss of vomeronasal neuron responsiveness to specific pheromones [14], and V1R (*V1Rb2*) specifically responds to the mouse pheromone, 2-heptanone [15]. Collectively, these results indicate that V1R functions as a pheromone receptor. Mouse V2R-expressing vomeronasal neurons specifically recognize exocrine gland-secreting peptide 1 [16]. They also respond to certain pheromone candidates, such as major histocompatibility complex ligand peptide [17] and mouse urinary protein [18]. These results suggest that V2R also functions as a pheromone receptor.

Putative functional V2R genes are present in mouse, rat, opossum [19] and platypus [20], but no functional V2R genes have been reported in dog, cow or human [19]. In contrast, intact V1R genes have been identified in a variety of mammals, including mouse, rat, opossum, dog, cow and human [20-22]. However, the V1R gene repertoire varies dramatically among different mammals [21,23]. The large and diverse V1R repertoire and species-specific delineation of certain V1R subfamilies, particularly in mice and rats [22,24,25], suggests that V1Rs contribute to species-specific recognition of pheromones and thus have played certain roles in rodent speciation. However, the intact V1R repertoire is much smaller in non-rodent mammals, such as cow and dog [21], and the roles of V1Rs in these animals are not well understood.

In general, pheromones have been thought to have species-specific functions [1]. However, in ruminants such as goats and sheep, a goat male pheromone, which induces out-of-season ovulation in anestrous females (known as the male effect pheromone), also affects sheep, and vice versa [26,27], indicating that this pheromone functions across species. Exposure of female sheep to the male effect pheromone stimulates c-fos expression in both the accessory olfactory bulb and main olfactory bulb [28], raising the possibility that this pheromone can be detected by both the VNO and the main olfactory epithelium (MOE). One goat V1R gene (*gV1R1*) is expressed in both the VNO and the MOE, and functional V2Rs have not been detected in goat [29]. Thus, goat V1R is a good candidate for detecting this male effect pheromone. Our current study shows that the V1R gene repertoire is quite conserved among goat, sheep and cow, and thus, all of the orthologous cow-goat-sheep V1Rs are predicted to detect the same or closely related chemical compound(s). Furthermore, all identified goat V1R genes were expressed both in the VNO and the MOE, suggesting that V1R-mediated chemical information is transmitted by the main and accessory olfactory pathways.

## Results

### Isolation of the goat and sheep V1R genes

Based on sequence similarity with 32 previously reported cow V1R genes [21], we identified 23 goat and 21 sheep V1R genes. Initially, we isolated these V1R genes using degenerate primers, which were designed based on conserved sequences present in each V1R subfamily among the various vertebrate species. Unexpectedly, all the sequences we obtained had high sequence similarity with the corresponding cow V1R genes. To obtain the complete coding sequences of the V1R genes, we searched the cow genome database  for flanking nucleotide sequences adjacent to the coding sequences of each of the cow V1R genes. We then designed primers (Additional file 1: Primers used to isolate the coding sequences of goat and sheep V1R genes.) and isolated the complete sequences of intact goat and sheep V1R genes from the respective genomic DNAs by PCR. We obtained a total of 24 intact goat V1R genes, including one that we previously isolated (*gV1R1*) [29]. Deduced amino acid sequences of the newly isolated V1Rs are listed in Additional file 2: Deduced amino acid sequences of newly isolated goat and sheep V1Rs.

### Conserved repertoire of orthologous V1R genes in cow, goat and sheep

**Figure 1:**
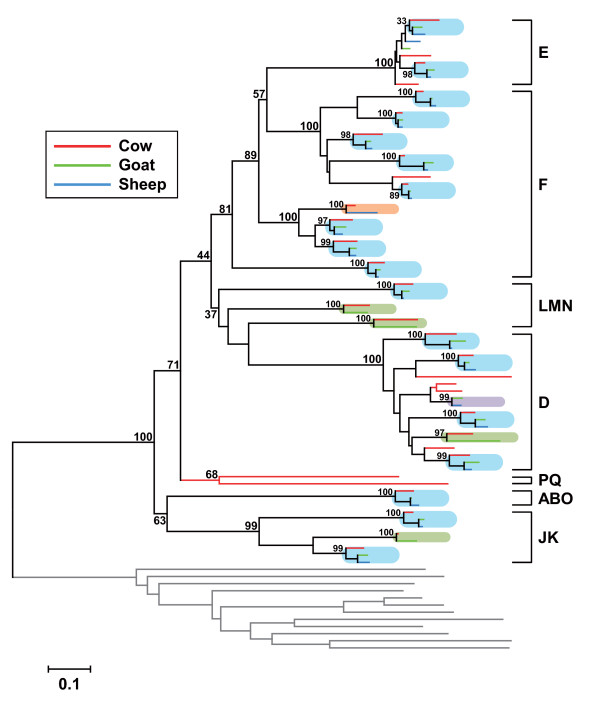
**Phylogenetic tree of intact V1R genes of cow, goat and sheep**. Analysis of 32 cow, 24 goat and 21 sheep V1Rs. Cow T2R genes (gray) [31] were used as the outgroup. Bootstrap percentages supporting the monophyly of each family group are given. The tree was reconstructed using the neighbor-joining method with Poisson-corrected protein distances. The tree includes the following V1R sets: Eighteen cow-goat-sheep (pale blue), four cow-goat (pale green), one cow-sheep (pale orange), and one goat-sheep (pale purple). The scale bar indicates 0.1 amino acid substitutions per site. The subfamilies are indicated on the right.

**Figure 2:**
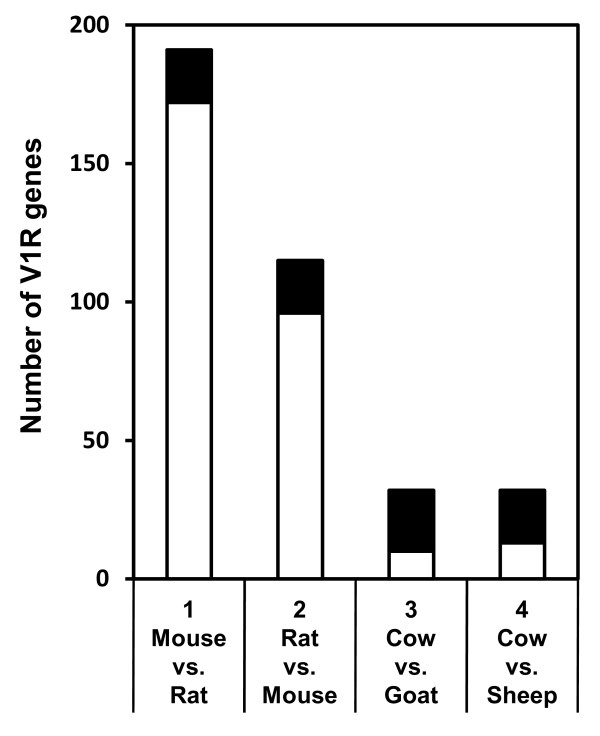
**The number of V1R genes having orthologs in other species**. The overall height of each bar indicates the total number of mouse (bar 1), rat (bar 2) and cow (bars 3 and 4) V1R genes. The black bars show the number of mouse-rat (bar 1), rat-mouse (bar 2), cow-goat (bar 3) and cow-sheep (bars 4) V1R orthologs.

A neighbor-joining tree of intact V1R genes derived from goat, sheep and other mammalian species [20,21,29,30] indicates that goat and sheep V1Rs cluster in the A/B/O, D-F, J/K and L/M/N subfamilies, which fall within previously described cow V1R subfamilies (A/B/O, D-F, J/K, L/M/N, P, Q) [21]. Figure 1 shows a neighbor-joining tree for cow, goat and sheep V1Rs. The cow taste type 2 receptors (T2Rs) [31] were used as the outgroup in this tree. All of the goat and sheep V1Rs have orthologs in cross-species ruminant counterparts. There were 18 sets of cow-goat-sheep, four pairs of cow-goat, one pair of cow-sheep, and one pair of goat-sheep orthologous V1Rs. Thus, 68.8% (22 genes) or 59.4% (19 genes) of the 32 cow V1R genes have orthologs in goat or sheep, respectively (Figure 2). These percentages are remarkably high compared with those corresponding to mouse and rat orthologs; only 19 mouse-rat orthologous V1R pairs were found among 191 mouse and 115 rat V1R genes, representing 9.9% of mouse (19 of 191) and 16.5% of rat (19 of 115) V1R genes, respectively (Figure 2). Thus, orthologous V1R genes exist at significantly higher rates in cow than in mouse and rat.

The complete goat and sheep genomic sequences are not available at present. Thus, we tried to estimate the approximate size of the goat and sheep V1R gene repertoire by genomic Southern blot analysis under conditions of low stringency. Genomic DNAs were digested with the restriction enzymes that do not cut inside the V1R genes, and the resulting fragments were size fractionated by agarose gel electrophoresis, transferred to the membranes, and hybridized with the probes. As probes, we selected one to three goat V1R genes from each subfamily, which should detect majority of the genes belonging to each subfamily under conditions of low stringency. (Additional file 3A: Genomic southern blot analysis of V1R genes, underlined V1Rs). We assume that the number of the generated bands corresponds to that of goat and sheep V1R genes in each subfamily. In the V1R genes subfamilies A/B/O, D, E/F, J/K, and L/M/N, we detected 2, 8, 15, 5, and 4 bands for goat, and 2, 8, 15, 4, and 5 for sheep, respectively (Additional file 3B, shown by the arrowheads). We detected a somewhat larger number of V1R genes in goat and sheep compared with cow (i.e., for cow: 1, 9, 14, 3 and 3 intact V1R genes for respective subfamilies above) [21], possibly a consequence of cross-hybridization of the probes with pseudogenes in the goat and sheep genomes. Hence, the approximate size of the repertoire of the intact V1R genes in goat and sheep might be similar to that in cow. These data suggest that the orthologous V1R genes exist at significantly higher rates in goat and sheep than in mouse and rat, although we cannot rule out the possibility that unidentified V1R gene families exist in goat and sheep genomes.

**Figure 3:**
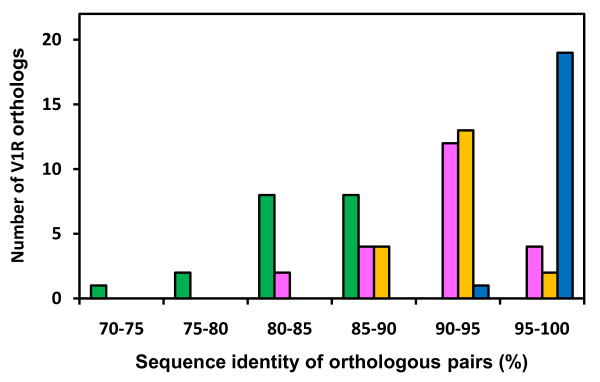
**Similarity analysis of orthologous V1Rs**. The number of orthologous V1R pairs in mouse and rat (green), cow and goat (magenta), cow and sheep (yellow), and goat and sheep (blue) at different levels of amino acid sequence identity is shown.

Next, we analyzed the identity of V1R orthologous pairs among goat, sheep and cow. We chose the 22 cow-goat, 19 cow-sheep, and 19 goat-sheep orthologous V1R pairs that clustered in monophyletic branches (Figure 1). We calculated and compared amino acid sequence identities for these pairs with those of the 19 mouse-rat orthologous V1R pairs (Figure 3). The identity in each set of orthologous pairs ranged from 80.6 to 97.1% for cow-goat, 87.5 to 97.4% for cow-sheep, and 94.2 to 99.1% for goat-sheep, whereas the corresponding identity ranged from 71.2 to 88.6% for mouse-rat orthologous V1R pairs. The average sequence identity was 91.2% for cow-goat, 91.8% for cow-sheep, and 97.1% for goat-sheep orthologous V1R pairs, and these values are substantially higher than the 83.9% for the mouse-rat pairs. These observations suggest that, among ruminants, the orthologous V1R pairs are quite similar each other at the sequence level.

### Expression of V1R genes in the goat VNO and MOE

**Figure 4:**
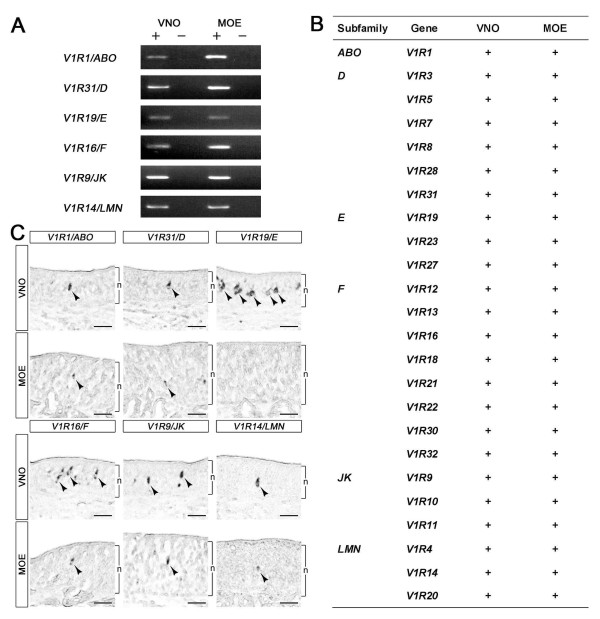
**Expression of goat V1R genes in the VNO and the MOE**. A: Expression of goat V1R genes in the VNO and the MOE was examined by nested RT-PCR. The cDNAs were synthesized with (+) or without (-) reverse transcriptase. Specific primers for detecting each V1R gene are listed in Additional file 4. B: Summary of the expression of goat V1R genes detected by nested RT-PCR analysis. All goat V1Rs identified thus far are expressed in the VNO and in the MOE (indicated by +). C: *In situ *hybridization analysis. Coronal sections from goat VNO or MOE were hybridized with antisense V1R probes. Arrowheads indicate V1R-expressing cells. "n" indicates the neuroepithelial layer. Scale bar indicates 50 μm.

One goat V1R (*gV1R1*) is expressed in both the VNO and the MOE [29,32]. We extracted RNAs from the VNO and the MOE to analyze the expression of the newly isolated V1R genes by nested reverse transcription-PCR (RT-PCR). We found that all V1R genes were expressed in the VNO and the MOE (Figure 4A and 4B). Although zone-specific expression of olfactory receptors has been reported in rodents [33,34], such a V1R expression pattern was not observed in goat MOE (data not shown). To obtain a more precise understanding of V1R gene expression, we performed *in situ *hybridization analysis and found that all V1Rs, except *V1R19*, are expressed in small subsets of the cells in the neuroepithelial layer of the MOE as well as in that of the VNO (Figure 4C and unpublished results) [29,32]. Because *V1R19 *was initially detected by RT-PCR analysis, failure to detect *V1R19*-expressing cells in the MOE might reflect the relatively small number of these cells. Collectively, our results suggest that all goat V1R genes identified thus far are expressed in both olfactory organs, the VNO and MOE.

## Discussion

We identified and analyzed goat and sheep V1R genes (Additional file 2) by comparison with 32 cow V1R genes in the cow genome database [21]. All the V1R genes we identified have orthologs in these cross-species ruminant counterparts (Figure 1). In cow, over 70% (23 of 32) of V1Rs have orthologs in goat and/or sheep (Figure 1 and 2). This is a surprisingly high percentage because in rodent only 9.9% of the mouse (19 of 191) and 16.5% of the rat (19 of 115) V1R genes have an ortholog in their cross-species counterpart. So, species-specific V1Rs, which represent 85-90% of mouse and rat V1Rs, are almost nonexistent in cow, and "conserved" V1Rs, which have orthologs among these ruminant species, have been retained (Figure 2). Thus, cow V1Rs are distinct from the species-specific V1Rs of rodents [24,25,30]. We speculate that the role of V1Rs in defining species specificity has degenerated in ruminants. Alternatively, the function of defining species specificity may have developed in rodent V1Rs after the ancestral separation of ruminants from rodents.

The amino acid sequence identity of cow-goat, cow-sheep and particularly goat-sheep orthologous V1R pairs (Figure 3) was remarkably high, strongly suggesting that, within each set/pair, these orthologous V1Rs detect the same or closely related chemical compound(s). We speculate that ruminant V1Rs might function to detect compounds that are evolutionarily conserved and/or commonly essential for the survival of these species. Therefore, the main function of ruminant V1R might differ from that of rodent V1R.

Expression of V1R genes in the MOE has been reported in lower vertebrates, such as fish [35] and frog [36]. However, such expression has been regarded as the exception in mammals, although V1R expression in the MOE has been reported in human [37] and goat [29]. Expression of all 24 goat V1R genes in both the VNO and the MOE (Figure 4) suggests that these V1Rs play a role in chemoreception in the MOE and that V1R-mediated chemical signals are likely transmitted by two morphologically distinct pathways. Similar parallel processing of certain pheromone and pheromone candidates in the main and accessory olfactory systems has been reported in rodents [38-40]. However, it is unlikely that rodent V1Rs are the receptor for these compounds in the MOE, because functional expression of V1Rs has not been reported in rodent MOE [9]. Therefore, analysis of V1R-mediated parallel processing systems in ruminants should provide a profound understanding of the diverse systems of mammalian chemoreception.

These features of V1R orthologs in goat and sheep, and the expression of the V1Rs in the MOE (Figure 4), raise the possibility that V1R(s) is involved in detecting the male effect pheromone. The male effect pheromone affects both goat and sheep [26,27] and seems to be detected by the MOE [28]. Studies have suggested that volatile chemicals are the candidates for the male effect pheromone [41,42]. Furthermore, the V2R-mediated signal transduction pathway, which is believed to play a role for pheromone reception in rodents, does not seem to be involved in the goat pheromone reception system [29,43]. Taken together, these data suggest that V1R is a very good candidate receptor for the male effect pheromone.

The expression of V1Rs in both olfactory organs, the MOE and VNO, may indicate the dual system for perceiving the pheromone information in goat; it appears likely that the presence of the male effect pheromone is first detected by MOE V1R, which induces the flehmen response to actively intake pheromone molecules into the VNO for more precise quantitative analysis with the V1R in it, and if the amount of pheromone is assessed to exceed a certain threshold level, the information is then conveyed into deeper part of the brain, resulting in eventual stimulation of the gonadotropin-releasing hormone pulse generator activity in the hypothalamus. Although studies have reported that the pulsatile secretory pattern of luteinizing hormone is accelerated without functional VNO under certain experimental conditions [44,45], this result does not preclude the potential importance of the VNO in mediating the pheromone effect under the natural social circumstances, where the levels of airborne pheromones are so subtle that a well-coordinated dual olfactory system needs to play a vital role in detecting as well as perceiving them. At present, we cannot rule out the possibility that other receptor families, such as the olfactory receptor, might also be involved in recognizing the male effect pheromone in the MOE. Thus, functional identification of the receptor(s) for the male effect pheromone requires further study.

## Conclusion

The repertoire of V1R orthologs is substantially more conserved among three ruminant species than between rodent species, raising the possibility that each orthologous ruminant set/pair detects the same or closely related chemical compounds. These results may explain the "inter-species" pheromonal effect between goat and sheep. In goat, all V1Rs identified thus far are expressed in both the VNO and the MOE, suggesting that in the MOE these V1Rs play a role in chemoreception and that V1R-mediated chemical signals are likely transmitted by two morphologically distinct pathways. These features of ruminant V1Rs seem to be distinct from those of rodent V1Rs, suggesting that the ruminant V1Rs may have evolved different functions than rodent V1Rs.

## Methods

### Animals and tissues

We used adult Shiba goats (*Capra hircus*) and Corriedale sheep (*Ovis aries*) that were maintained in the National Institute of Livestock and Grassland Science (Tsukuba, Japan). Goats were sacrificed by an overdose of pentobarbital (30 mg/kg; Dainippon Pharmaceutical Corporation, Osaka, Japan). The VNO and the MOE were rapidly removed and used for RNA extraction, or tissues were fixed in phosphate-buffered saline (PBS, pH 7.4) containing 4% paraformaldehyde overnight at 4°C for histological analysis.

### Identification of V1R homologs

Based on the homology of cow V1R genes [21], V1R homologs were isolated from goat or sheep genomic DNAs by PCR using the primers listed in the Additional file 1. Genomic DNAs were obtained from goat liver as described [29] and sheep blood. To prepare the genomic DNA from the blood samples, packed blood cells were mixed with a buffer containing 72 mM NH_4_Cl and 8 mM NH_4_HCO_3 _for 20 min and centrifuged for 5 min at 4°C. The pellet was washed in the same buffer 2-3 times, dissolved in a buffer containing 40 mM Tris-HCl (pH 7.4), 0.1 M NaCl, 10 mM EDTA, 400 mg/ml proteinase K and 0.5% SDS, and incubated overnight at 50°C. The DNA was purified by two cycles of phenol extraction and precipitated with ethanol. The DNA was dissolved in distilled water and used as a DNA template for PCR. PCR amplification (35 cycles) was carried out at 95°C for 1 min, 55-60°C for 1 min, and 72°C for 1 min. Amplified DNA fragments were cloned into a pBluescript SK vector (Stratagene, La Jolla, CA, USA).

### Sequence alignment and phylogenetic analysis

ClustalX version 2.0.5 [46] was used for multiple protein alignments with manual adjustment. Amino acid sequence-based phylogenetic trees were reconstructed using the neighbor-joining method [47] with Poisson-corrected protein distances [48] and were evaluated by 1,000 bootstrap replications. MEGA4 [49] was used to assess evolutionary relatedness. The identity (%) of the amino acid sequences of the orthologous V1Rs was calculated using the EMBOSS pairwise alignment algorithms [50].

### Identification of orthologous V1R genes

Mouse-rat orthologous V1R genes were identified among 191 mouse and 115 rat V1R genes [30] as described by Grus and Zhang [24]. Briefly, we identified clades in the phylogenetic tree that contain a single mouse and a single rat gene and then calculated the synonymous substitutions per site (d_S_) between the putative orthologs. Those with an estimated d_S _within the range of 0.119-0.316 were accepted as orthologs. The same method was applied to cow-goat, cow-sheep, and goat-sheep orthologs. The estimated d_S _ranges were 0.039-0.147, 0.030-0.119, and 0.014-0.065, respectively.

### Southern blot analysis of goat and sheep genomic DNA

Ten micrograms of goat and sheep genomic DNAs were digested with the restriction enzymes, which do not cut inside the coding sequences of the V1R genes. The digested genomic DNAs were size fractionated by agarose gel electrophoresis, blotted on nylon membrane (Biodyne Plus, Pall, MI, USA), and hybridized under conditions of low stringency. The probes were prepared and labeled with digoxigenin (DIG) using PCR DIG probe synthesis kit according to the manufacturer's instructions (Roche). Hybridization was carried out in a solution containing 30% formamide, 7% SDS, 5 × SSC, 0.1% N-lauroylsarcosine, 50 mM phosphate buffer, pH7.0 and 2% blocking reagent (Roche, Mannheim, Germany) at 42°C overnight, followed by relaxed washing with 2 × SSC containing 0.1% SDS at 55°C. Hybridized probes were detected by using an alkaline phosphatase-conjugated anti-DIG Fab fragment antibody and CDP-Star according to the manufacturer's instructions (Roche), and visualized using LAS4000 IR multi color (Fuji film, Tokyo, Japan).

### RT-PCR

Goat total RNAs were extracted from the VNO and the MOE using PureLink Micro-to-Midi Total RNA Purification System (Invitrogen, Paisley, UK) and dissolved in RNase-free water (0.03-0.2 μg/μl). RNA (0.15-1.0 μg) was treated with DNase in 50 μl reactions containing 50 mM Tris-HCl (pH 7.5), 10 mM MgCl_2_, 10 mM NaCl, 10 mM DTT (Invitrogen), 1 μl RNase inhibitor (Wako, Osaka, Japan) and 4 μl DNase I (Promega, WI, USA) at 37°C for 20 min. The DNA-free RNAs were extracted by phenol-chloroform (1:1), precipitated with ethanol, dissolved in 20 μl RNase-free water, and stored at -80°C. The cDNAs were synthesized by SuperScript III (Invitrogen) according to the manufacturer's instructions, using purified RNAs as templates and CTGATCTAGAGGTACCGGATCC (dT)_24 _as the primer at 50°C for 1 h followed by inactivation of the enzyme at 70°C for 15 min. The cDNAs synthesized without SuperScript III were used as the negative control. To perform nested PCR, the first PCR amplification (35 cycles) was carried out at 95°C for 1 min, 55-60°C for 1 min, and 72°C for 1 min with the cDNA products as templates. The deoxynucleotide primers used included CTGATCTAGAGGTACCGGATCC along with those listed in Additional file 4: Primers used for nested RT-PCR analysis. The second PCR was carried out with the first PCR product as template with the primers listed in Additional file 4 at 95°C for 1 min, 55-60°C for 1 min, and 72°C for 1 min (35 cycles). The second PCR products were electrophoresed and visualized using ethidium bromide.

### In situ hybridization

Adult goat VNO and MOE were sectioned coronally (6 μm thick), and sections were hybridized with DIG-labeled cRNA probes as described [51]. Briefly, sense and antisense DIG-labeled riboprobes were synthesized with a DIG RNA labeling mix according to the manufacturer's instructions (Roche, Basel, Switzerland). The sections were fixed with 4% paraformaldehyde/PBS for 5 min, immersed in 0.3% H_2_O_2_/PBS for 15 min, and treated with 0.5 μg/ml proteinase K/PBS for 10 min. The sections were then neutralized in 0.2% glycine/PBS for 5 min, immersed in 0.2 N HCl for 20 min, treated with 0.1 M triethanolamine for 15 min, and washed with PBS for 1 min. Hybridization was carried out overnight at 55°C in a hybridization solution containing 50% formaldehyde, 1 × Denhardt's solution, 10 mM Tris-HCl pH 7.6, 0.2 mg/ml yeast tRNA, 0.6 M NaCl, 0.25% SDS, 1 mM EDTA and 10 μg/ml of DIG-labeled cRNA probe. Hybridized sections were washed in 5 × SSC at 50°C for 30 min, 50% formaldehyde in 5 × SSC at 50°C for 30 min, followed by treatment with 2 μg/ml RNase (Sigma, St. Louis, MO, USA) at 37°C for 30 min. Samples were then washed twice in 2 × SSC at 50°C and twice with 0.2 × SSC at 50°C. After treatment with blocking reagent (Roche) for 30 min, sections were incubated with an alkaline phosphatase-conjugated anti-DIG Fab fragment (Roche) overnight at 4°C. Positive signals were visualized using 4-nitro blue tetrazolium chloride and 5-bromo-4-chloro-3-indolyl-phosphate (Roche) as chromogenic substrates.

## Competing interests

The authors declare that they have no competing interests.

## Authors' contributions

HOh carried out the molecular genetic studies. MN carried out the sequence alignment. AD participated in the molecular genetic studies. KM participated in the histological studies. HOk participated in preparing animals and histological studies. NO provided the phylogenetic information for mammals. YT participated in data analysis. YM participated in the design of the study. KH conceived the study, supervised the study, and finalized the manuscript. All authors read and approved the final manuscript.

## Supplementary Material

Additional file 1 Primers used to isolate the coding sequences of goat and sheep V1R genes. Click here for file

Additional file 2 Deduced amino acid sequences of newly isolated goat and sheep V1Rs. Click here for file

Additional file 3 **Genomic southern blot analysis of V1R genes**. A: Phylogenetic tree of intact V1R genes of goat and sheep was reconstructed as described in Materials and Methods. The V1R genes underlined were used as subfamily-specific probes. B: Goat (lines 1, 3-4, 7-8, 11-12, 15-17) and sheep (lines 2, 5-6, 9-10, 13-14, 18- 20) genomic DNAs were digested with EcoRI (lines 1-2 and 7-10), HindIII (lines 3-6 and 11-14) or BglII (lines 15-20). The digested DNAs were electrophoresed on 0.8% agarose gels, blotted on nylon membranes, and hybridized with DIG-labeled probes under conditions of low stringency. Goat V1R1 (lines 1 and 2), V1R31 (lines 3 and 5), V1R8 (lines 4 and 6), V1R30 (lines 7 and 9) and V1R19 (lines 8 and 10), V1R10 (lines 11and 13) and V1R9 (lines 12 and 14), V1R14 (lines 15 and 18), V1R4 (lines 16 and 19), and V1R20 (lines 17 and 20) were used as probes. The hybridized probes were removed from the membranes (lanes 3, 5, 7, 9, 11, 13, 15 and 18), and each membrane was re-hybridized with another V1R gene of the same subfamily (lanes 4, 6, 8, 10, 12, 14, 16 and 19), respectively. The arrowheads indicate the generated bands. Click here for file

Additional file 4 **Primers used for nested RT-PCR analysis**. The first PCR was carried out using the sense primers, as listed, and the antisense primer, CTGATCTAGAGGTACCGGATCC. The second PCR was carried with the primers listed. Click here for file
