# Supplementary material for: Conserved repertoire of orthologous vomeronasal type 1 receptor genes in ruminant species
Source: BMC Evol Biol. 2009 Sep 15;9:233. doi: 10.1186/1471-2148-9-233 (PMC2758851; doi:10.1186/1471-2148-9-233)
Supplement: Additional file 1 — Primers used to isolate the coding sequences of goat and sheep V1R genes. [file 1471-2148-9-233-S1.pdf]

V1R gene	Sense Primer (5'-3')	Antisense primer (5'-3')
V1R1	AGACTTTCCAGTTCCATTGACATAAG	CATATGGGTGAGATGGCATTAA
V1R3	TGAGAAACCCAGGGGAGATG	TGAAATAGTAAATATGAAGC
V1R4	GTCCCATAGATACCAATATG	TAACCAGTTGACTCTATTCT
V1R5	AATCCCCATTCAGAAAGATG	TGAATCTTGGCAAATAGCTG
V1R7	CTCTTGACATCCTCTCCATG	GACTTGTTAGAATATATGG
V1R8	ACCACCTATCTCTTACAGATG	CCTGAATTATTAAAATGTATTG
V1R9	AGAGAGACTGAAAGACAATG	CTCCGTAGTATTCTGCACCA
V1R10	AAAACGGGATTAAGATAATG	TGACCTGATACCCGCAGGCA
V1R11	CGAAGGAAAACATATAAAAC	TGATCTGATGCTTGTATCTT
V1R12	TCTTGAGACCTGAGAGGATG	GTCCTAGGTAAAATTAT
V1R13	GCTCACATACTCCTGCAATG	ATTTTCCACTTGTATTCACA
V1R14	GTTTTCTCTAAAGATAACTC	CTTCTGTGAAAGAAATTAAA
V1R16	CACTGAGACCTGACGGCATG	TAATGTCCAACTTTGTGTGA
V1R17	GACTGTGAACCGGGAGGATG	TGAATTGGGTGTATTTCTGA
V1R18	GTTGCCTTTCAGTTTACATG	AAATGGTATATATTTGCAC
V1R19	AAGAAAGTTGTCTCTCGATG	TGAAGAACTCAGTATCCCCT
V1R20	TGACTTGAAGAAATTCATACA	CCTCATTCTATGAAGTACCT
V1R21	GACTGTGAACTGAGAGGATG	TGAGCCACTGCCAGCTCAT
V1R22	TACTCAGACCTGACAGCATG	TAAATGGTATGTGTTTGGCT
V1R23	AGGAAACTTGGCTGTTGATG	CAAGTTATACTTGATTTCTC
V1R27	AGGAAAGTTGGCTGTCAATG	CCACGCACCTATGGACACCTT
V1R28	TGGTGGAGAATAAGGAAATG	GATGCCTTCAATAATAACCA
V1R30	CACTGAGACCTGAGAGCATG	TGAATGGTATGTATTTGCAC
V1R31	CTCTGACATCCTCTCCATG	GAGTCATTAGAATGTATGA
V1R32	GACTGTGAACTGAAAGGATG	ATCATTGCAGCGTGGTTC
