# Supplementary material for: Conserved repertoire of orthologous vomeronasal type 1 receptor genes in ruminant species
Source: BMC Evol Biol. 2009 Sep 15;9:233. doi: 10.1186/1471-2148-9-233 (PMC2758851; doi:10.1186/1471-2148-9-233)
Supplement: Additional file 2 — Deduced amino acid sequences of newly isolated goat and sheep V1Rs. [file 1471-2148-9-233-S2.pdf]

>goatV1R3
MVLETISLLQMVVGAPGNVILFFHSICPIWLGRKTRPTEVILAHMVVSN
LLIIFSPGIPHIIVAFVLRKPLSSLGCKFVYYVQRVARGTTLCSTCVLSTY
QSFTLIPRRAEWVMLRGRAPRVIGPSCCACWLLSFLMNICVPVTVAGP
QGTNNYTDNHGKWFCSSSAPKARFLYLWSVSDAVFIALMVWSSGSM
VLLLLRHRQRVQYIHTPTGRHRRPPETRAAHTILMLVVTFVTFYALDCI
FAFRIAAFLDFRLSLLHTANILASCFPTVSPLLLLLRDPRTHGCCSWVFR
HRG
>goatV1R4
MMASSKWEMGIILVTQMGVGILGNSFLLCFYNFTLLTGCKVRPTDLIL
NQLVLANSLVLFSRGIPHIMATFGSRNFLSEAGCKFLFYFQRVARGICL
SMTSLLSAFQAMRLCPHFSSWLGLRTRSSKFIGSCVFLSWILHLLVNISI
ILYITSPTHNESYSIKVNYGYCSSLDVARNVRFVCPAIFFTMDFICLSLM
VWASGSMVVFLLRHKQRVQHIHSNSHSPRACAEARAASTILVLASSFC
SLYSLSALLSIWMSLFAIPGQWLVDASVFLSLCFPTVSPFVLICSDTRVT
RKTGLSSWR
>goatV1R5
MLCPQKESGEMFLKALFLFQVGNGALADVTLFFCSVSPALLGHKRRP
LQTTVAHMAVANSWVLLSAGPPHTMAAFVSRKPLSSLGCEFVYSVQR
VALSTALCSTCVLSTYQSFTLTPRRAEWVMLRGRAPTVTGPSCCTCW
MLSLLMNIWIPVSISGPQDKHNYTDAQGMWFCSPSASKAGFVYLWST
SDAVFLTLMVCSSGSTVLLLHRRRQRVQYVHTPAGHHRSPPETRAAH
TTLMLVVTFVTFHLLNSVLTFYNRAHDDFRLWLMQLSDALALCFPTV
SPFLLLLRDPRTPRFCS
>goatV1R7
MFFHKDALRTTSQAALKITYLTQMGIGSLVNVILFFHSISPVLIGQSQKP
TDMIHTHMAVANLLVLLSPGIPHTMAASFPRKPLSGLGCKFVYYIQRV
ARSTALCSTCILSTYQSFTLTPRRAEWVMLRGRAPKVIGPSCCTCWML
SFIMHIIAPLKITGPPNMHNYTDTKDKWFCSSSPDEIGTSYLWSVSDAV
FIGLMVWSSGSMVLLLLRHRQRVQYIHTPAGHHRCPPETRAAHTILML
VVTFITFYFLNSVLSFYIAAFFDIRLWLIQTSNALGSCFPTICPFLLLLRD
PRTPRFCS
>goatV1R8
MGVGSLVNVILFFQNIFPVLFGHKQRPTDTILTHMALANLLVLLSSGIP
HTMTAFVSSKPLSSLGCKLVYYIHRVARSTTLCSTCVLSTYQSFTLIPRS
EGRALLRRACRVTDSSCWICWIFSALVNIYIPVNITGPQDTHNYSDTQG
RWFCTSLGLKSGFTFLWSVSDAIFISLMIWSSGSMVLLLHRHHQKMKY

IHTRTGHHRCPPETRATHTILMLVVTFVIFYVLNSVFAFCISAFHDFRL
WLLQTSSVLVSCFPTVSPFLLLLRDPRAPRFSS
>goatV1R9
MISSDKILGIFFFFQICIGLMGNSLLFILYMYIFLILPHKKKPTDVILTHLT
FANTLTLVFRGVPNILLSFGISPKMGDIGCKAVIYIQRVTRSISLYTTSLQ
STFQAVTITLSNSKWVWLKIKFSTFIQPSLLFSWIINMVIYSEIILRNVAN
RNITDARSGYYAAYCKTDVPHHHIVATFLSAIFTRDFFLLSLMTCSSIY
MVNILFRHRKTAQHVRSTIQSSQGAPEIKAINFILMLVSCFVFFYWTNT
FLTVYLFSVSGNNWQLESFGNYVASCYPSICPFLLMKNENRISRINYIK
TRIRIFSF
>goatV1R10
MLASDAILEFFFMSQICIGLMGNSVLFKLKMCTCITQPHLKKPIDVIFIH
LTLVNVLTIMFKLIPDVMSSFGVRHFLDDVGCKATLFTHRVTRGLSICT
TALLSAYQAITISPVHSKWVWLKSKLSTCIYPSFLFFWVINMLIYIHIIKT
VVANLNFTTVGSGYSTLYCQTNQLEHHYYSMAFLSVILIRDLLFVSLM
ACSSLYMVTLLYKHRRRALHIHSPTLSPRPAREIQATHVILWLVSCFVF
FYFSDNFITLYLFYRHEKNWSLKRMNGIISSCYPMICPFVLMKNNKIVS
KCISSISKMRMAFSRRTLKR
>goatV1R11
MFSSDAILGFFFISLICIGLMGNLVLFTLYIDTFLTQSCLKKPIDVIFIHLT
LVNVLTILFKLIPDVMPSFGERYFLDDVGCKTTLYIYRVTRGLSICTTAF
LSVFQAITINPVNSKWAWLKSKLSMCIYSSFLFFWVINLLIYIHIIETVR
ARNNFTTAGLGYYAVYCQTEQLEHHYSMAFLNVMVIRDLLFVVLMM
WSSIYMVTLLYKHRQRAQHLHSLSLSPQSSPEIKATHTILLLVGCFAFF
YRLNNFIAFYLLYSPKKKPEMERITGIISSCYPSLCPFVLMTKKMSQLTS
FLSRVTITFSQRTFSR
>goatV1R12
MAAGDLTAGFILLLQTVFGMLGNFSLLYHYLFLYCTGIRLKSIDLLVK
NLIVANILVLLSCGFHNIVANFQWHHLDRDFACRFFPYVRVVGRGVSI
GTTCLLSVFQVITISPRNSKWAGLKVKALKYVVPSIILCWIVNMLVNVI
YLMFLSGDLSNKSITNRKRYGHCSSVRHDQTRDSLFAALLSFPDVVCF
VVMIWASGSTVLILYRHKQRVQNIHSIKISSISSPESRATKTILLVVSTFV
NFNTLSTICNIVLSLLNSPSLFFVNSSAIVTACFPTISPFLIMSRDSRISRLC
FAEKRNTNSPTPRRKM
>goatV1R13
MPSYSEHKPWGLGGGGVASRDLAVGVISLSQTVVGILGNFLFLYQNV
TIHLRGHRLKPLDFILRHLIFANILVLLSKGLPQMVAAFGLRHFLSDPG

CKLIFYVHRVAAGVSFSTTCLLSVLQAIMISPRNSTWTSLKGKAPKYIS
FILYLCWVLHLLVNTFVFTYVSGKWSSMNTTKQKDVGYCSITHLDTIT
STLLAAFLSPPSTLSFGFMTLASGYMVFTLYRHHQQVRHIPRTNRSAGS
PPETRAAQRILALVSTFLSFYMLCTIFVTYMLVFNHPSWWLINISSLTRS
CFPTVSPFLLMSPDTYVSRLCSACCRRNNPVPNWVRKL
>goatV1R14
MDYIDLTLRIFFLSQTGIGLLGNSFLFLSIFTFHAGDKVRPSDHIPKHIFL
ANSLVLLSRGIPQTMVSFGWNNFLDDHGCKFLFYIHRVAREVCLSSTC
LLSIFQAITIIPNKSRWAELKARVPKYIGPLCFLCWVVLFLGNILVPLKV
TGPKRIRNITLSNFGYCSGARLHPITSPLYAVFHAFIDMMCLGLLVWTS
SSMVLYLYTHRQQVKYIHSSRHISRHSPEKKATQNILILVCTFVLFFTLS
SISSIYMAVFNIPSWWLMNANVFLDACFPTFCPFVFISGHIHISKLCFVF
CERN
>goatV1R16
MAARDLAVAVIFLSQTIFGMLGNFSVLYHYFFLCFTGQRLRTIDLIVKN
LIVANILVLSSSGFHDTVKNFEWHFMDSDFACRFFPYVRVVGRGVSIG
TTFLLSVFQAITISPRTAKWAELKVKALKCMVPSVILCWTLNMLVNVI
YPMYMSGTLRNRSTTNRKDLGHCSAVSHGQPEDSLYAVLLSFPDALC
FVLMILASGSTVFILYRHKQRVQNFRRTNISTISSPESRATKTILLLVSTF
ICFNILTFICTIIFSVFDSIDVFFVKTSAVIIACFPTVSPFLLMSRDARISRN
CFAGIRHSISPTLICAKSLQ
>goatV1R18
MVPVFHISCSHSGPSSSEDTHQSLRPDSMAAGDSEIRTVFLSQTLFGVL
GNVSLLYHYLFLYCTGCRLRTIDLIVKNLIVPKILVLFSNGYHYTMTNY
GWHHMDSDFTCRFSPYARGVSRGVPIGTTFLLSVFQAITICPRTSRWEE
LKVKALQLVFPSIILCWIVNMLGNVVYPMYVTGNLSNKNFTNRKRFG
HCFSVRHDQIRDSLYAALISFSDVLCFVVTILASGSTAFILYRHKQRLK
NFHRIKVSSISSPESRATKTVLLVVSTFVCFNTLFSIYYIVLSVLKDLDLF
IMNISAIITACFPAISPFLLMSRDSRISRLCFAGKRNTNSPTLMRKM
>goatV1R19
MSQIYMSHSGLRNHCCHHIKGSILNDTTASSQLAFGITFLLQTVLGIWG
NFSLLYRYFFLYHTQCRLRVTDLICKHLTIANILVILSKGVPQTITTLRL
KYFASDFACKLILYVERVGRTMSISTTCLLSVFQTITISPKNSCWKNLK
VKAPKYIAFSISFCWIQRMFVHLFFPLYALYVSEKWHSTNMTNTRDSG
YCAATDLENISGSIYTALVVFPEVSFSVLIFWASGSMILTLYRHSQRVQ
YIHKASVSPRLPAESRATQSILLLASTFMCFHTLSCIFNITLALFHNPSW
WLVYTTGLINVCFPFISPFLLMSRDSIISSLCLLYMKNSVSP

>goatV1R20
MISVDGAIGTVILAQAGIGTAGNVSLLCHYICSLFKRHGLRPIEQIINHL
ALANTLTLICGAIPPTLAAFEMKYFLSDIGCKLVFYFHRVAWGTSLSVT
CLLGGFQALSINPPNSKWAELKFKSQKYITTPCILSWMFHLLVNVIVPM
RMTGQKNGRNVSVKSNLGHCSCLSINAITESICSVIFSSVDVVCLGFMI
WVSGSMILFLHRHKQQVQHIHSTRQPSPTSPETRATKSIMLLVSAFVIF
YFLSSAFEMYAYLFDNPQLWLVNTSVVLASSFPTLSPFLLLKSNTHVSS
LCSSC
>goatV1R21
MAGSFFIIGMIILTQTVLGILGNFSLLCSYIVLHVTGYRLRSTDLILKHLI
VANSLVLLCKGVPQTVAVFGWKHICSDFGCKLLFFLHRVGRGVSICSI
CLLSVFQVITISPWNSRWAVLKVTAPKYTVPSICLCWILQMLVNVIFPI
YITGKWSHNNITEERDFGCCSTILTDQKNKKTKDALYAALLSFPDVLC
LGLTLWAGGSMVLILYRHKQQVQHIRRTDASSRSSPESRATKTILLLG
GTFVYFYTLSSIFQVLLALFVQPSWFFVNMTVIIAAWF
>goatV1R22
MAAGDLAAGFILLSQTVVGMLGNFSLLYHYLFLYCTGYRLRTVDLIV
KNLIVGNILVLFSAGFHSTMTNFGWNHLNSGSACRFFAYVRGVGRGT
SIGITCILSVFQAIMISPRNSKWAKLKVQALKFIVPSIFLCWILNLLVNLN
YPIFVTRIMNNKSITNRKSFRHCTAIHHDRSGDIFIAMMFFPVVLCFLL
MIWTSGSTVFILYRHKQRVQNFHRISVSSISSAESRATKTILLLVSIFVIF
NTLSSISYIVLGLLNNPSLFISTMSSIITSYFPTVSPFLLMSRDPTISRLCFA
GKRNTNAPTPMRKM
>goatV1R23
MSQIYMSHSALKNHCCHHLKGIILNDTIASSQLAIGIIFLLQTVLGIWGN
FSLLYHYLFLYHTLCRLRVTDLICKHLTIANFLVILSKGVPQTITTLRLK
YFASDFACKLILYVERVGRSMSIGTTCLLSVFQTITISPKNSCWKNLKV
KAPKYIAFSISFCWIQCMFVNLFFPLYALYVSGKWHSTNMTNTRDSGY
CVATDLENISGSIYIALVVFPEVSLSVLIFWASGSMILTLYRHSRQVQYI
HKACVSPRSSAESRATQSILLLASTFMCFHTLSSIFNITLALFHNPSWWL
VYTTGLINVCFPFISPFLLMSRDSIVSSLCFLYMKNTKSPNLNRKT
>goatV1R27
MSQIYINHPALRHHCCHHLKGSILNDTTASSQLAIGIIFLLQTVLGIWGN
FSLLYHYLFLCHTQYRMKVTTLICKHLAIANFLVILSKGVPQTITTLRL
KHFANDFACKLILYVERVGRSMSIGTTCLLTVFQTITISPINSYWKNLKI
KAPKYIAFSISFCWIQCMFVHLIFPLYALYVSDKRHSTNMRNTRDSGY
CSDIDLEKILGSIYVALIVFPEISFSVLIFWASGSMILTLYRHSQRVQYIH

KAIVSPRSSAESRATQSILLLASTFMCFHTLSCIFNICFALYHNPSWWLV
YTTGLINVCFPFISPFLLMSRDSIVSSLCFLYMKNSVLHNLIRKM
>goatV1R28
MSFHKDALRDAGEATVKTIFLFQVVVGTLGNAILFSRSISPVLLGHKQ
RPTLMVLPHLALANLLALLSPGIPHIMAAFVSRKPLSSLGCKFVYYIQR
MALSTALCSTCVLSTYQSFTLTPRRAEWVMLRGRAPKVTGPSCCTCW
MLSLLMYIPVPLKITGPQDTHNYTDSQSNWFCSISGTVTSVGYLWFISD
AVFLTLMVWSNGSMVLFLHRHHQRVQYIHTPTGHHRRHPETRATHTI
LMLMVTFIIVYILNSTFSFYLNVLVEFRLWLMQTSDALASCFPTVSPFL
LLLRDPKTPRICSGVNRNDA
>goatV1R30
MATGDLAAGSILLLQTVFGMLGNFSLLYCYLFLCCTGDRLKTVDLIVT
NLIVANICILFSTGFCSPITTFGWNCLKSGSACRVFAYLRGVGRGASIGI
TCILSVFQAITISPRNSRWAELKVKALKCVVPSIILCWVVNMMLNVIYP
IFVTGILSNKSITNRKSFQHCSAVPNDRYGETYAAMMSMPVVFSFVVM
ILASGSTVFTLYRHKQRVQNVHGINGSSISSAESRATKTILLLMGIFISFN
TLSSISYIILGISNNAGLFISTMSAVVISCFPAISPFLLMSRDSRVSRLCFA
GERNANSPTLKRKV
>goatV1R31
MSLHKDALRTTSQTALKTTYLIQTGVGSMANIILFLHNISPILLGHNQR
PTPTILAHISLANLLFLLSSGIPHIMTAFVLGNPLSSLGCKFVYYIQRVAR
STALCSTCVLSTYQSLMLTPRRAEWVMLRGKALKVTGPSCCTCWMFS
LLMYIYVPVKITGPWDRYNNSDSQGKWFCSISGTVTVFGYLWFISDA
MFITLMVWSSGSTVLLLHRHHQRVQYLHTPAGHHRCPPETRAAHTIL
MLMVAFVTFYLLNYSLVFHISASSDFRLWLLQVSNILVSCFPTIFPFLLL
LRDPRTPRFCS
>goatV1R32
MAGSFFIIGMIILTQTVVGILGNFSLLCSYIILHVTGYRLRSTDLILKHLI
VANSLVLLCKGVPQTMAVFGWKHILSDFGCKLLFFLHRVGRGVSIGSI
CLLSVFQVITISPRSSRWAVLKVTAPKYMVLSLFLCWILQMLVNVIFLF
HITGKWSDKNITKEKDFGYCSSGLTDKTQHALHAALLLFPDVLCLGLT
LWAGSSMVLILYRHKQQVQRIRRTNASSRSSPESRATKTILLLGSTFVY
FYILSSICQVLWLFLISPAGSLWISL
>sheepV1R1
MKKNRLSSSIDIRNAIFSEVAIGILANAILLLFHAHNFLLEHRPKSTDLTI
GNLALIHIVMLLTVAFMATDTFGSQKTWDDIQCKLVVYLYSLMRGLSI
CATCLLSVLWAITLSPRNSRLAKFKLRPSHHNLYCTLFLWVFNMFING

SFLLSTIATPNVTSAHLLRVTESCSLRPVIHFLRYVQFVLRTIQDICLLGL
MALSSGYMVTLLYRHKRQTQHLQSTKLSPKASPGERATQTILFLMSFF
VVMYFLDITVSWFSRMLWDIDSVRMCVQMLVGNGYASISPLVLISTE
KRIIKVLKYIWVRWH
>sheepV1R3
MVLETISLLQMVVGALGNVILFFHSICPTLLGRKTRPTEVILAHMVVSN
LLIIFSPGIPHIIVAFVLRKPLSSLGCKFVYYVQRVARGTTLCSTCVLSTY
QAFTLTPRRAEWVMLRGGAPKVIGPSCCACWLLSFLMNICVPVTVTG
PQGTNNYTDNHGKWFCSSSAPKARFLYLWSISDAVFIALMVWSSGSM
VLLLLRHRQRVQYIHTPTGRHRRPPETRAAHTILMLVVTFVTFYALDCI
FAFRIAAFLDFRLSLLHTANILASCFPTVSPLLLLLRDPRAQGCCSWVF
RHRG
>sheepV1R7
MFFHKDALRTTSQAALKITYLTQMGIGSLVNVILFFHSISLVLIGQSQRP
TDMIHTHMAVANLLVLLSPGIAHTMAASFPRKPLSGLGCKFVYYVQR
VARSTALCSTCVLSTYQSFTLTPRRAEWVVLRGRAPRVIGPSCCTCWV
LSFIMHITAPLKITGPPNMHNYTDTKDKWFCSSSPDEIGTSYLWSVSDA
VFIGLMVWSSGSMVLLLLRHHQRVQYIHTPAGHHRCPPETRAAHTIL
MLVVTFITFYFLNSVLSFYITAFFDIRLWLIQTSNALGSCFPTICPFLLLL
RDPRTPRFCS
>sheepV1R8
MGVGSLVNVILFFQNIFPVLFGHKQRPTDTILTHMALANLLVLLSSGIP
HTMTAFVSSKPLSSLGCKLVYYIHRVARSATLCSTCVLSTYQSFTLIPR
SEGRSLLRRARRVTDSSCWICWLFSALVNIYIPVNITGPQDTHNYSDTQ
GRWFCTSLGHKSGFTFLWSVSDAVFISLMIWSSGSMVLLLHRHHQKM
KYIHTRTGYHRCPPETRATHTILMLVVTFVIFYVLNSVFAFCISAFHDF
RLWLLQTSSVLVSCFPTVSPFLLLLRDPRAPRFCS
>sheepV1R9
MISSDKIFGIFFFFQICIGLMGNSLLFILYMYIFLILPHKKKPTDVILIHLTF
ANTLTLVFRGVPNILLSFGISPKMGDIGCKAVIYIQRVTRSISLYTTSLQS
TFQAVTITLSNSKWVWLKIKFSTFIQPSLLFSWIINMVIYSEIILRNVANR
NITDARSGYSAAYCKTDAPHHHIVATFLSAIFTRDLFHLSLMTCSSIYM
VNILFRHRKTAQHVRSTIQSSQGSPEIKAINFILMLVSCFVFFYWTNTFL
TVYLFSVSGNNWKLESFGNYIASCYPSICPFLLMKNENRISRINYIKTRI
RIFSF
>sheepV1R11
MFSSDAILGFFFISLICIGLMGNLVLFTLYIDTFLTQSCLKKPIDVIFIHLT

LVNVLTILFKLIPDVMPSFGERYFLDDVGCKTTLYIYRVTRGLSICTTAF
LSVFQAITINPVNSKWAWLRSKLSMCIYPSFLFFWAINLLIYIHIIGTVR
ARNNFTTAGLGYYAVYCQTEQLEHHYSMAFLNVMVIRDLLFVVLVM
WSSIYMVTLLYKHRQRAQHLHSLSLSPQSSPEIKATHTILLLVGCFVFF
YCLNNFIAFYFLYLPKKNPEMERITGIISSCYPSLCPFVLMKNKMSQLTS
FLSRVTITFSQRTF
>sheepV1R12
MAAGDLTAGFILLLQTVFGMLGNFSLLYHYIFLYCTGIRLKSIDLLVKN
LIVANILVLLSCGFHNIVANFQWRHLDRDFACRFFPYVRVVGRGVSIG
TTCLLSVFQVITISPRNSKWAGLKVKALKYVVPSIVLCWIVNMLVNVI
YIMFLSGDLSNKSITNRKRYGHCSSVRHDQTRDSLHAALLSFPDVVCF
VVMIWASGSTVLILYRHKQRVQNIHSIKISSISSPESRATRTILLVVSTFV
NFNTLSTICNIVLSLLNSPSLFFVNSSAIVTACFPTISPFLIMSRDSRISRLC
FAEKRNTNSPTPRRKM
>sheepV1R13
MPSYSEHKPWGLGGGGVASRDLAVGVISLSQTVVGILGNFLFLYQNV
TIHLRGHRLKPLDFILRHLIFANILVLLSKGLPQMVAAFGLRHFLSDPG
CKLIFYVHRVAAGVSFSTTCLLSVFQAIMISPRNSTWTSLKGKAPKYTS
FILYLCWVLHLLVNTFVFTYVSGKWSSMNTTKQKDVGYCSITRLDTIT
STLLAAFLSPPSTLSFGFMTLASGYMVFTLYRHHQQVRHIPRTNRSAGS
PPETRAAQRILALVSTFLSFYMLCTIFVTYMLVFNHPSWWLINISSLTRS
CFPTVSPFLLMSPDTYVSRLCSACCRRNNPVPNWVRKL
>sheepV1R16
MAARDLAVAVIFLSQTIFGMLGNFSVLYYYFFLHFTGQRLRTIDLIVKN
LIVANILVLSSSGFHDTVKNFEWHFMDSDFACRFFPYVRVVGRGVSIG
TTFLLSVFQAITISPRTAKWAELKVKALKCMVPSVILCWTLNMLVNVI
YPMYMSGTLRNRSTTNRKDLGHCSAVSHGQPEDSLYAVLLSFPDALC
FVLMILASGSTVFILYRHKQRVQNFRRTNISTISSPESRATKTILLLVSTF
IFFNILTFICTIIFSVFDSIDVFFVKTSAVIIACFPTVSPFLLMSRDARISRN
CFAGISHSISPTLICAKSLQ
>sheepV1R17
MASIDLTIGVIFLMQMVLGIPGNFSLLCHHIILHFTGSRLRSTDLILKHLI
VANSLVLLCKGVLQTMAVFGWKHIRSDFGCKLLFFLHRVGRGVFIGSI
CLLSVFQVVMISPWNSRCAALKVKTPKYIVPSIFLHWILQMLVNFIFPL
HINGKWNDKNNANKKDFGYCSSIHPDKIQQSLSAALLSLPDVLCLGF
MLWASSFMVFILYRHKHRVQHIRRTDSPTKFSPETRATKTILLLVSTFV
YFYNLSSIFQLMMALFDYPSWFLVNITVILALYVPTVSPFLLMSCDYN

VHRLYFAWIRNTKSPTIMRNV
>sheepV1R18
MVPVFHISCSHSGPSSSEDTHQSLRPDSMAAGDSEIRTVFLSQTLFGVL
GNVSLLYHYLFLYCTGCRLRTIDLIVKNLIVPNILVLFSNGCHYTMTNY
GWHHMDSDFTCRFFPYARGVSRGVPIGTTFLLSVFQAITICPQTSRWEE
LKVKALQLVFPSIILCWIVNMLGNVVYPMYMTGNLSNKNFTDRKRFG
HCFSVHHDQIRDSLYAALISFSDVLCFVVTILASGSTGFILYRHKQRLK
NFHRIKVSSISSPESRATKTVLLVVSTFVCFNTLFSIYYIVLSVLKDPDLF
ITNISAIIIACFPAVSPFLLMNRDSRISRLCFAGKRNTNSPTLMRKM
>sheepV1R19
MSQIYMSHSGLRNHCCHHIKGSILNDTTASSQLAFGITFLLQTALGIWG
NFSLLYRYFFLYHTQCRLRVTDLICKHLTVANILVILSKGVPQTITTLRL
KYFASDFACKLILYVERVGRSISISTTCLLSVFQTITISPKYSRWKNLKV
KAPKYIAFSISFCWIQCMFVNLFFPLYALYVSEKWHSTNMTNTRDSDY
CAAADLENISGSIYAALVVFPEVSFSVLIFWASGSMILTLYRHSQRVQY
IHKACVSPRLPAESRATQSILLLASTFMCFHTLSCIFNTTLALFHNPSW
WLVYTTGLINVCFPFISPFLLMSRDSIISSLCFLYMKNSVSP
>sheepV1R20
MISVDGAIGTVILAQAGIGTVGNVSLLCHYICPLFKRHGLRPIEQIINHL
ALANTLTLICGAIPPTLAAFEMRYFLSDIGCKLVFYFHRVAWGTSLSVT
CLLGGFQALSINPPNSKWAELKFKSQKYITTPCILSWMFHLLVNVIVPM
RMTGQKNGRNVSVKSNLGHCSCLSINTITESICSVIFSSVDVVCLGFMI
WVSGSMILFLHRHKQQVQHIHSTRQPSPTSPETRATKSIMLLVSTFVVF
YFLSSAFEMYAYLFDNPQLWLVNTSVVLASSSPTLSPFLLLKSNTHVSS
LCSSC
>sheepV1R21
MAGSFFIIGMIILTQTVVGILGNFSLLCSYIILHITGYRLRSTDLILKHLIV
ANSLVLLCKGVPQTMAVFGWKHIRSDFGCKLLFFLHRVGRGVSICSIC
LLSVFQVITISPWNSRWAVLKVTAPKYTVPSLFLCWILQMLVNVIFPIYI
TGKWSHNNITEERDFGCCSTILTDQKNKKTKDALYAALLSFPDVLCLG
LTLWAGGSMVLILYRHKQQVQHIRRTDTFSRSSPESRATKTILLLGGTF
VYFYTLSSIFQVLLALFVQPSWFFVNMTVITAACFPTVSPFLLMSRDSS
VHRLYFAWMRNAKSSTIMRKA
>sheepV1R22
MAAGDLAAGFILLSQTVVGMLGNFSLLYHYLFLYCTGYRLRTVDLIV
KNLIVGNILVLFSAGFHSTMTNFGWNHLNSGSACRFFAYVRGVGRGT
SISITCILSVFQAITISPRNSRWAKLKVQALKFIVPSIFLCWILNLLVNLN

YPIFVTRIMNNKSITNRKSFRHCTAIHHDRSGDIFIAMMFFPVVLCFLL
MIWTSGSTVFILYRHKQRVQNFHRISVSSISSAESRATKTILLLVSIFVIF
NTLSSVSYIVLGLLNNPSLFISTMSSIITSYFPTVSPFLLMSRDPTISRLCF
AGKRNTNAPTLMRKM
>sheepV1R23
MSQIYMSHPALKNHCCHHLKGIILNDTIASSQLAIGIIFFLQTVLGIWGN
FSLLYRYLFLYHTLCRLRVTDLICKHLTIANFLVILSKGVPQTITTLRLK
YFASDFACKLILYVERVGRSMSIGTTCLLSVFQTITISPKYCRWKNLKV
KAPKYIAFSISFCWIQCMFVNLFFPLYALYVSEKWHSTNMTNTRDSEY
CAATDLEKISGLIYTALIVFPEVSFSVLIFWASGSMILTLYRHSRQVQYI
HKACVSPRSSAESRATQSILLLASTFMCFHTLSSIFNITLALFHNPSWLL
VYTTGLINVCFPFISPFLLMSRDSIVSSLCFLYMKNTKSPNLNRKT
>sheepV1R27
MSQIYINHPALRHHCCHHLKGSILNDTTASSQLAIGIIFFLQTVLGIWGN
FSLLYHYLFLYHTQYRMRVTTLICKHLAIANFLVILSKGVPQTITTLRL
KHFANDFACKLILYVERVGRSMSIGTTCLLTVFQTITISPINSYWKNLKI
KAPKYIAFSISFCWIQCMFVHLIFSLYALYVSDKSSSTNMRNTRDSGYC
SDTDLEKISGSIYVALIVFPEISFSVLIFWASGSMILTLYRHSQRVQYIHK
AIVSPRSSAESRATQSILLLASTFMCFHTLSSIFNICFALYHNPSWWLVY
TTGLINVCFPFISPFLLMSRDSIISSLCFLYMKNSVLHNLIRKM
>sheepV1R28
MSVHKDALRNAGEAAVKTIFLFEVVVGALGNAILFSCSISPVLLGHKQ
RPTQMVLPHLALANLLALLSPGIPHIMAAFVSRKPLSSLGCKFVYYVQ
RMALSTALCSTCVLSTYQAFTLTPRRAEWAMLRGRAPKVTGPSCCTC
WMLSLLMYIPVPLKITGPQDTHNYTDSQGNWFCSISGIVTSVGYLWFIS
DAVFLTLMVWSSGSMVLFLHRHRQRVQYIHTLTGHHRRHPETRATHT
ILMLMVTFIIVYILNSTSSFYLTVLVEFRLWLMQTSDALASCFPTVSPFL
LLLRDPKTPRICSGVNRIDT
>sheepV1R30
MATGDLAAGSILLLQAVFGMLGNFSLLYCYLFLCCTGDRLKTVDLIVT
NLIVANIFILFSTGFCSPITTFGWNCLKSGSACRVFAYLRGVGRGASIGI
TCILSVFQAITISPRNSRWAELKVKALKCVVPSIILCWVVNMMLNIIYPI
FVTGILSNKSITNRKSFQHCSAVPNDRYGETYAAMMSMPVVFSFVVMI
LASGSTVFTLYRHKQRVQNVHRINGSSISSAESRATKTILLLMGIFISFN
TLSSISYIILGISNSAGLFISTMSAVVISCFPAISPFLLMSRDSRVSRLCFA
GKRNANSPTLKRKV
>sheepV1R31

MSLHKDALRTTSQTALKTTYLIQTGVGSMANIILFLHNISLILLGHNQR
PTPTILAHISLANLLFLLSSGIPHIMTAFVLGNPLSSLGCKFVYYVQRVA
RSTALCSTCVLSTYQSFTLTPRRAECVMLRGKAPRVTGPSCCTCWMLS
LLMYIYVPVKITGPWDRYNNTDSQGKWFCSISGTVTGFGYFWFISDA
MFITLTVWSSGSTVLLLHRHHQRVQYLHSPAGHHRCPPETRAAHTILM
LMVAFVTFYLLNYSLVFHISASSDFRLWLLQVSNILVSCFPTIFPFLLLL
RDPRTPRFCS
>sheepV1R32
MAGSFFIIGMIILTQTVVGILGNFSLLCSYIILHVTGYRLRSTDLILKHLI
VANSLVLLCKGVPQTMAVFGWQHIRSDFGCKLLFFLHRVGRGVSIGSI
CLLSVFQVITISPRSSRWAVLKVTAPKYMVLSLFLCWLLQMLVNVIFPF
HITGKWSDKNITKEIDFGYCSSGLTDKAQHALHAALLLFPDVLCLGLT
LWAGSSMVLILYRHKQQVQHIRRTDASSRSSPESRATKTILLLGSTFVY
FYILSSIFQVLWLFLISPAVSLWISL
