# Supplementary figures and images for: Conserved repertoire of orthologous vomeronasal type 1 receptor genes in ruminant species
Source: BMC Evol Biol. 2009 Sep 15;9:233. doi: 10.1186/1471-2148-9-233 (PMC2758851; doi:10.1186/1471-2148-9-233)

**A**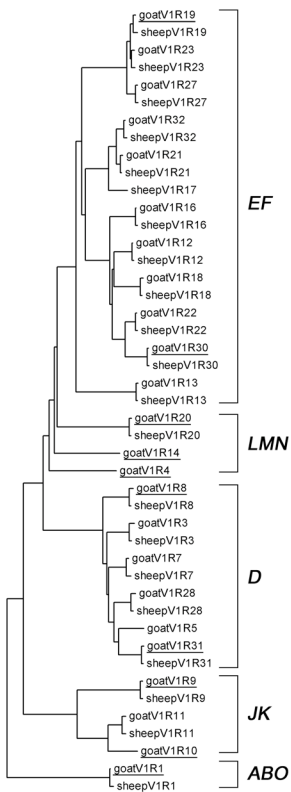**B**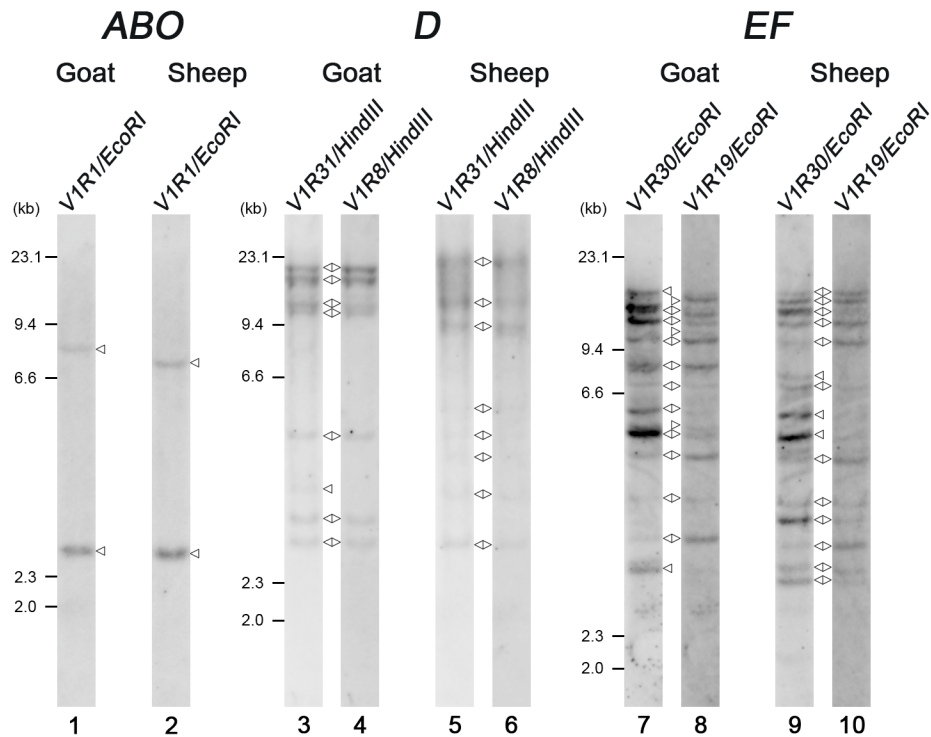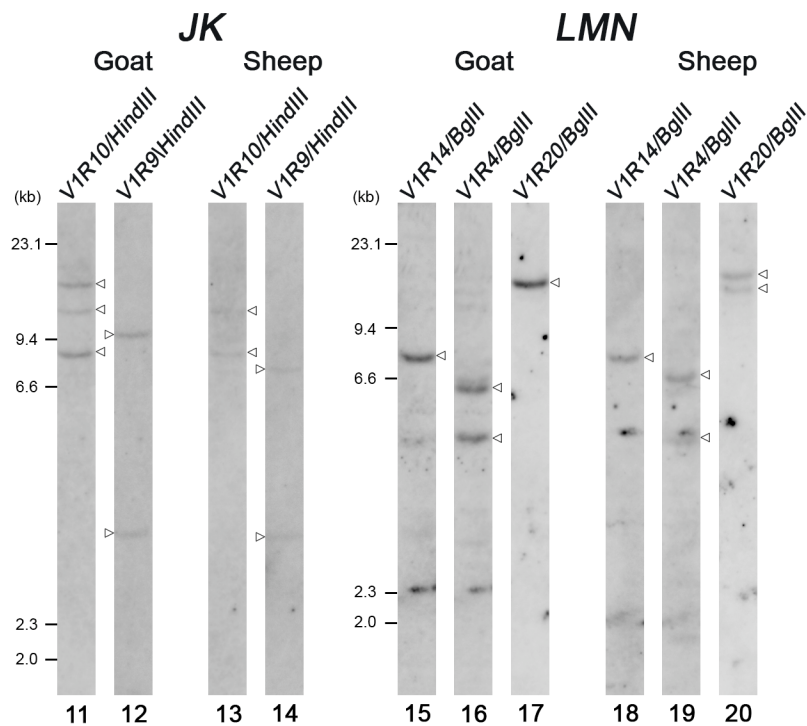

Supplement: Additional file 3 — Genomic southern blot analysis of V1R genes. A: Phylogenetic tree of intact V1R genes of goat and sheep was reconstructed as described in Materials and Methods. The V1R genes underlined were used as subfamily-specific probes. B: Goat (lines 1, 3-4, 7-8, 11-12, 15-17) and sheep (lines 2, 5-6, 9-10, 13-14, 18- 20) genomic DNAs were digested with EcoRI (lines 1-2 and 7-10), HindIII (lines 3-6 and 11-14) or BglII (lines 15-20). The digested DNAs were electrophoresed on 0.8% agarose gels, blotted on nylon membranes, and hybridized with DIG-labeled probes under conditions of low stringency. Goat V1R1 (lines 1 and 2), V1R31 (lines 3 and 5), V1R8 (lines 4 and 6), V1R30 (lines 7 and 9) and V1R19 (lines 8 and 10), V1R10 (lines 11and 13) and V1R9 (lines 12 and 14), V1R14 (lines 15 and 18), V1R4 (lines 16 and 19), and V1R20 (lines 17 and 20) were used as probes. The hybridized probes were removed from the membranes (lanes 3, 5, 7, 9, 11, 13, 15 and 18), and each membrane was re-hybridized with another V1R gene of the same subfamily (lanes 4, 6, 8, 10, 12, 14, 16 and 19), respectively. The arrowheads indicate the generated bands. [file 1471-2148-9-233-S3.pdf]
