# Supplementary material for: Conserved repertoire of orthologous vomeronasal type 1 receptor genes in ruminant species
Source: BMC Evol Biol. 2009 Sep 15;9:233. doi: 10.1186/1471-2148-9-233 (PMC2758851; doi:10.1186/1471-2148-9-233)
Supplement: Additional file 4 — Primers used for nested RT-PCR analysis. The first PCR was carried out using the sense primers, as listed, and the antisense primer, CTGATCTAGAGGTACCGGATCC. The second PCR was carried with the primers listed. [file 1471-2148-9-233-S4.pdf]

V1R gene	1st PCR	2nd PCR	
	Sense Primer (5'-3')	Sense Primer (5'-3')	Antisense primer (5'-3')
V1R1	TAATCCACATAGTCATGCTA	TGGGATGACATCCAATGTAA	ATGCTTGTGGGAAATGGCTA
V1R3	TCCTGAGCACCTATCAGTCC	CTGTTGCGCCTGCTGGTTGC	GCCCTGATGGTCTGGTCCAG
V1R4	TAACTTCACTTTGCTCACTG	CAGACTTGATTCTCAATCAG	TGGCCAGAGGGATTTGCCTT
V1R5	TCCTGAGCACCTATCAGTCC	TCCTGAGCACCTATCAGTCC	GGCCCATGATGATTTTCGCC
V1R7	TCCTGAGCACCTATCAGTCC	GTTCTGTTCATCCTCACCTG	CGTCTATGGCTGATACAGAC
V1R8	TCCTGAGCACCTATCAGTCC	TCCCTAGAAGTGAAGGGAGG	GGTGGTTCTGCACGTCCTTG
V1R9	ATGTCTTTGGGCTAATGGGGAA	TCTTTTTAATTCTTCCCCATAA	TTATGGGGAAGAAGTGGGCC
V1R10	ATGTCTTTGGGCTAATGGGGAA	ACTTGCATAACTCAGCCTCA	GCCTATCAAGCCATCACCAT
V1R11	ATGTCTTTGGGCTAATGGGGAA	CACTTTCTTAACTCAGTCTTG	AATTCTAAGTGGGCCTGGCT
V1R12	TACTGTGCTGGATTGTGAAC	TTCTGAGTGGAGATTTGAGC	TACAGCCACAAGCAAAGCGT
V1R13	CTGTAGTCGGAATCTTAGGG	ACGTCACCATTCACCTCAG	GCTCTGCACCATTTTTGTG
V1R14	GGAGACAAGGTGAGACCTTC	TCTTTAGTCCTTCTCTCTAG	GCAATTTTGGATACTGTTCT
V1R16	MTGDTRAATSTMAWTTATCC	ACATTGAGAAACAGAAGCAC	TCCACGGTTTTCATCCTGTA
V1R18	TACTGTGCTGGATTGTGAAC	GCTGGGAAATGTTGTGTATCC	CTGTCCTTCTTGTGGTGAGC
V1R19	CAGTGCATGTTTGTACATCTC	TTTCTGAAAAATGGCACAGC	TACAGCTTTGGTCGTGTTCC
V1R20	TCTGTTGATGGGGCAATAGG	ATCCCTCCAACATTGGCAG	GCAAGTCCAACACATTCACAG
V1R21	TAGCATCTGCCTCTTGAGTG	AGGTGATCACAATCAGTCCC	GACGCCTTGTATGCAGCATT
V1R22	MTGDTRAATSTMAWTTATCC	TGACTCGAATAATGAACAAC	GGCTCCACAGTTTTCATCTT
V1R23	ATGACACAATAGCCTCCAGC	CCTCTTTCTTTACCACACTCT	TACCCAAAGCATCCTACTC
V1R27	TCCTTCTGCTGGATCCAGTG	CCTCTATATGCATTATATGT	GTACAGTACATACACAAGGC
V1R28	TCCTGAGCACCTATCAGTCC	GTCTCTTAATGTACATCCCTG	GTTTCGTCTGTGGTTGATGCA
V1R30	MTGDTRAATSTMAWTTATCC	CGTGACTGGAATATTGAGTA	GGCTCCACAGTTTTCACTCT
V1R31	TCCTGAGCACCTATCAGTCC	GAAAATCACTGGTCCTTGGG	ACACCATCCTGATGCTCATG
V1R32	TAGCATCTGCCTCTTGAGTG	AGGTGATCACAATCAGTCCCC	TGGCAGCTCCATGGTTCTCAT
